# A Case of Challenging Polyarteritis Nodosa With Critical Limb Ischemia

**DOI:** 10.7759/cureus.97257

**Published:** 2025-11-19

**Authors:** Linn Renken, Caroline Zollinger-Read, Annabel Coote

**Affiliations:** 1 Rheumatology, Doncaster Royal Infirmary, Doncaster, GBR

**Keywords:** critical limb ischaemia, epididymo-orchitis, major limb amputation, peripheral vascular disease (pvd), polyarteritis nodosa, vasculitis

## Abstract

Polyarteritis nodosa (PAN) is a systemic vasculitis with necrotizing inflammation of medium and small-sized arteries. Critical limb ischemia is a rare complication of PAN. We present a case report of a 64​-year-old male who presented initially with constitutional symptoms, cutaneous lesions and bilateral orchitis. After admission, he developed critical ischemia of his right lower limb with milder ischemia in his other limbs. After failure to respond to intravascular thrombolysis, he ultimately required a right below-knee amputation. He had symptoms and signs of mononeuritis multiplex and developed clinical and radiological features of severe bilateral lower limb myositis. A diagnosis of PAN was suspected, and he was commenced on treatment with high-dose corticosteroids, pulsed intravenous cyclophosphamide, intravenous immunoglobulin and intravenous heparin. His recovery was complicated by a pontine stroke and a myocardial infarction. An angiogram of the right lower limb showed arterial occlusion below the knee. A CT angiogram showed splenic and renal infarcts. The ulnar arteries were occluded in the proximal forearms. His C-reactive protein was 344 at its peak and fell to <5 with treatment.

Our case report highlights an aggressive systemic presentation of PAN with extensive organ involvement including critical lower limb ischemia, cutaneous vasculitis, orchitis, mononeuritis multiplex, myositis and infarcts affecting brain, myocardium, kidneys and spleen.

## Introduction

Polyarteritis nodosa (PAN) is a vasculitis primarily affecting small and medium-sized arteries, inducing necrotising inflammation and thrombosis [1]. The term ‘nodosa’ reflects the ‘pearl necklace’ appearance of fibrous nodules along affected arteries, which gave the condition its name [2]. Once considered either primary or hepatitis B virus (HBV)-related, it is now recognised in association with haematological malignancies and genetic syndromes [2], and HBV vaccination has significantly reduced HBV-related cases [1]. PAN affects 0.9-8.0 per million annually in Europe [3] and is most often diagnosed between ages 40 and 60 [4].

The clinical presentation is often heterogeneous. Patients often initially present with symptoms including unintentional weight loss, loss of appetite, fever and night sweats [2,5]. PAN can affect any organ but preferentially targets the skin, the nervous system and gastrointestinal tract [2]. One aim of this article is to expand on the clinical presentations of PAN. Differential diagnoses of PAN include infective endocarditis, human immunodeficiency virus, and other vasculitides, such as granulomatosis with polyangiitis. It typically does not present with glomerulonephritis and there is a lack of anti-neutrophil cytoplasmic antibodies (ANCA), which helps distinguish PAN from other vasculitides [2]. Untreated PAN carries a three-month mortality of up to 50% [6], making timely diagnosis and initiation of immunosuppression crucial.

## Case presentation

A 64-year-old male presented to the Emergency Department with systemic symptoms, testicular swelling, bilateral lower limb weakness and ulcerative lesions on his lower limbs. He was a lifelong non-smoker and had a medical background of autoimmune hepatitis, confirmed with liver biopsy and currently treated with maintenance azathioprine. Seven years previously he had been diagnosed with a primary membranous glomerulonephritis, which had gone into remission after treatment with corticosteroids and oral cyclophosphamide.

The patient had returned from a cruise in Southeast Asia two months prior to this presentation. The patient reported a five-week history of intermittent fevers, night sweats, reduced appetite, generalised myalgia and arthralgia. He had developed ulcerative lesions on his lower limbs, which were unsuccessfully treated in the community as suspected infected insect bites with oral antibiotics. A few days prior to presenting to hospital, the patient reported worsening bilateral lower limb weakness, paraesthesia and testicular pain and swelling.

On admission, the patient’s C-reactive protein (CRP) was 344 mg/L. He was treated by the urologists for suspected epididymo-orchitis with broad-spectrum intravenous antibiotics. A couple of days after admission, the patient developed a critically ischaemic right foot and a poorly perfused left foot. An angiogram of the right lower limb showed occlusions of the anterior tibial, posterior tibial and peroneal arteries in the mid leg, but no aneurysms. The vascular surgeons treated him with intravascular thrombolysis and intravenous heparin, which was later changed to argatroban due to possible heparin resistance.

The testicular pain and swelling persisted, and the right lower limb ischemia worsened. The CRP remained >300 mg/L. On day 5 of admission, the opinion of a rheumatologist was sought due to the multi-systemic clinical features and unclear aetiology.

The rheumatologist noted the presence of vasculitic ulcers on his lower limbs and bilateral lower limb weakness with patchy sensory loss. A diagnosis of PAN was suspected based on the systemic symptoms, orchitis, vasculitic skin lesions, presumed mononeuritis multiplex and possible myositis. Treatment with intravenous methylprednisolone was commenced immediately and the patient received 1g on three consecutive days. A skin biopsy of the vasculitic lesions was not performed as it was deemed unlikely to change the immediate clinical management.

Laboratory investigations showed mild normocytic anaemia, normal renal function, raised creatine kinase and elevated acute phase reactants. The values are summarised in Table 1. The autoimmune workup included a weakly positive homogenous antinuclear antibodies (ANA) and negative ANCA. Other autoimmune investigations including double-stranded DNA, antiphospholipid antibodies and myositis autoantibody panel were all negative. Blood-borne viruses screen (HIV, hepatitis B, and hepatitis C), QuantiFERON, serial blood cultures and urine culture were negative. Computed tomography (CT) of chest, abdomen and pelvis did not identify any malignancy. Magnetic resonance imaging (MRI) of the spine showed degenerative spinal disease. MRI of both femurs revealed diffuse swelling of all muscle groups in the thighs, consistent with severe bilateral myositis.

**Table 1:** Serum investigations Data obtained from the patient's medical records (Doncaster Royal Infirmary, 2025). HIV: human immunodeficiency virus, ANA: antinuclear antibody, ANCA: antineutrophil cytoplasmic antibody, anti-GBM Ab: anti-glomerular basement membrane antibody, dsDNA: double-stranded DNA.

Serum investigation	Patient value and units	Reference range
Haemoglobin	131 g/L	126 – 180
C-reactive protein	344 mg/L	<5.00
Erythrocyte Sedimentation Rate	72 mm	1 – 20
Sodium	137 mmol/L	133 – 146
Potassium	4.0 mmol/L	3.5 – 5.3
Urea	3.5 mmol/L	2.5 – 7.8
Creatinine	44 umol/L	64-104
Estimated Glomerular Filtration Rate	>90 ml/min/1.73^2^	>90
Creatine Kinase	2033 IU/L	40 - 320
Serial blood cultures	Negative	
Hepatitis B surface antigen	Negative	
Hepatitis C antibody	Negative	
Malaria	Negative	
HIV	Negative	
ANA	Weakly positive, homogeneous	
ANCA	Negative	
Anti-GBM Ab	Negative	
dsDNA	Negative	
QuantiFERON	Negative	
Antiphospholipid Ab	Negative	
Myositis Ab screen	Negative	

A request was sent to the general surgeons for a quadriceps muscle biopsy. The vascular surgeons thought the patient would need a right lower limb amputation but agreed to delay the surgery in case the ischemia improved with treatment of his vasculitis. Nerve conduction studies were not requested due to relative contraindications, the patient’s anticoagulation and limb ischemia.

Pulsed IV cyclophosphamide at a dose of 15mg/kg was commenced and high-dose oral corticosteroids were given after the 3g of IV methylprednisolone.

The patient’s systemic symptoms and muscle strength began to improve and his CRP fell to 10 mg/L. However, the ischemia of his right foot and ankle worsened and he developed mild ischemia in both hands with ongoing similar symptoms in the left foot. Despite the anticoagulation and treatment with corticosteroids and cyclophosphamide, he had further thrombotic events. He complained of diplopia and a CT scan of his brain showed a pontine infarct. He also had a myocardial infarct with signs of anterior cardiac ischemia on an electrocardiogram, and a troponin I of 742 ng/L. Transthoracic echocardiogram reported impaired left ventricular systolic function with a left ventricular ejection fraction in the range of 36-49%. There was no cardiac thrombus visible on echocardiogram.

A CT angiogram showed multiple splenic and renal infarcts. The ulnar arteries were occluded in the proximal forearms. Due to the multiple infarcts and vascular compromise of the right hand, a further three consecutive daily doses of methylprednisolone 750mg were given. The patient was transferred to the Department of Critical Care so he could be monitored closely.

There was a delay in obtaining a muscle biopsy, partly due to his unstable clinical condition. Despite the initial treatment with corticosteroids and cyclophosphamide, the patient had persistent muscle weakness and signs of severe bilateral myositis on MRI, so a decision was made to administer IV immunoglobulin, through the NHS Clinical Commissioning policy for refractory Idiopathic Inflammatory Myopathies (IIM) [7].

**Figure 1:**
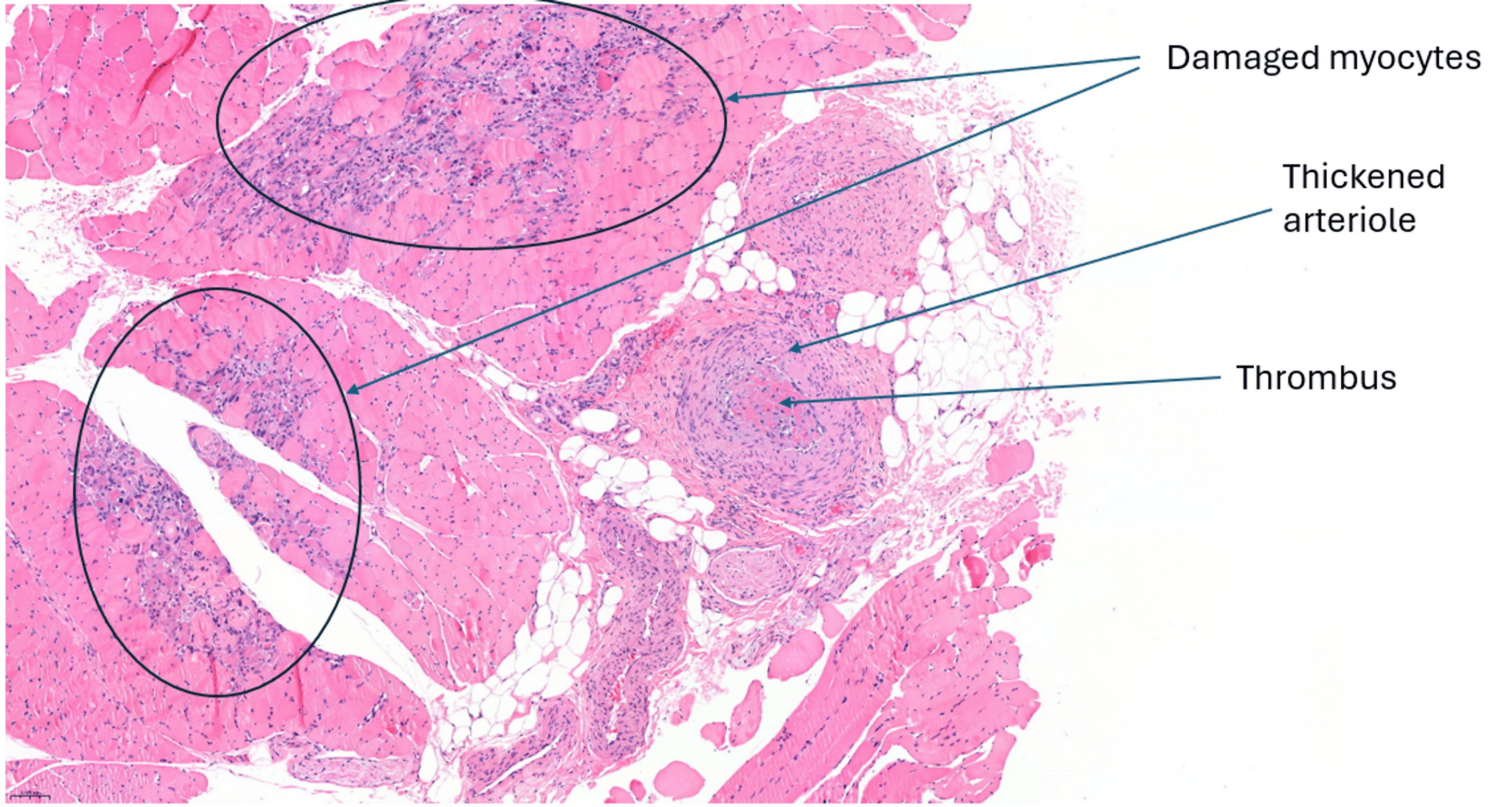
Right quadriceps muscle biopsy with Haematoxylin and Eosin stain, depicting damaged myocytes due to local ischemia and thickened arteriole containing a thrombus.

**Figure 2:**
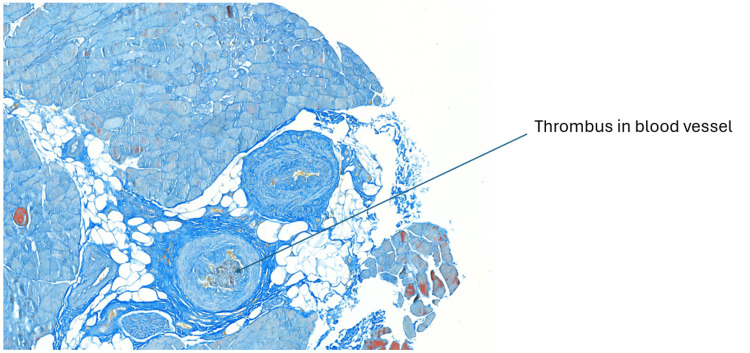
Right quadriceps muscle biopsy with Martius Scarlet Blue stain, depicting a thrombus within the blood vessel.

The ischemia of the right foot, ankle and lower leg persisted and was clearly demarcated. On day 21 of the patient’s admission, a vascular surgeon performed a quadriceps biopsy, immediately followed by a right below-knee amputation. Right quadriceps muscle biopsy showed no evidence of vasculitis or myositis (See Figures 1, 2). This was done at least three weeks after initiation of immunosuppression.

The patient made an excellent clinical recovery, with complete resolution of the systemic symptoms, cutaneous vasculitis and orchitis. His amputation wound healed well. He had fluctuating mild ischemic symptoms in his hands and left foot. His neuropathic symptoms slowly improved. In the postoperative period, he was treated with a therapeutic dose of dalteparin which was later switched to apixaban on the advice of the haematologists. The patient was transferred to a rehabilitation unit where he spent a few weeks prior to discharge. He was in hospital for a total of three months.

It is now six months after the patient’s hospital admission. He has had nine infusions of cyclophosphamide and is taking 2.5mg of oral prednisolone which he will stop soon. The plan is to treat him with a final infusion of cyclophosphamide (10 in total) before initiating treatment with mycophenolate mofetil. He has a lower limb prosthesis and is able to stand and mobilise with a walking frame. Clinically, the circulation in his hands and left foot has improved, but is still not normal. All his other symptoms have fully resolved.

## Discussion

PAN is a rare systemic necrotising vasculitis that predominantly affects middle-aged males. Signs and symptoms of PAN are attributable to vascular inflammation and ischemia of affected organs. The commonest organ manifestations are nerve involvement with mononeuritis multiplex followed by skin, gastrointestinal manifestations and kidney involvement. Systemic symptoms, arthralgia, myalgia and orchitis are commonly reported findings. The skin manifestations in PAN can include palpable purpura, nodules, livedo reticularis and ulceration, mainly on the legs.

There is no standard diagnostic test for PAN and diagnosis is based on the clinical picture, together with typical angiographic and biopsy findings. Histopathology of PAN is characterised by segmental transmural vectorising arteritis in medium-sized muscular arteries with features of fibrinoid necrosis and polymorphonuclear infiltrate [8]. Angiography usually reveals multiple micro aneurysms affecting the renal, hepatic and mesenteric arteries. ANCA is typically negative.

Our case satisfied five out of the 10 points of the American College of Rheumatology 1990 classification criteria for PAN [9]: weight loss >4kg, testicular pain, myalgia, mononeuropathy and arteriographic abnormality. Our patient initially presented with constitutional symptoms (weight loss and night sweats), testicular pain and skin lesions, which were misdiagnosed as infected insect bites. The patient proceeded to develop rapidly progressive critical limb ischemia and mononeuritis multiplex with high inflammatory markers.

Peripheral vascular disease (PVD) is the main differential of critical limb ischemia and is usually associated with older age or risk factors for atherosclerosis, such as smoking and diabetes mellitus. If PVD appears in a younger patient or in an older patient in the absence of risk factors, other causes should be considered such as vasculitis. There are a few case reports of PAN presenting as limb ischemia or rapidly progressive intermittent claudication in the literature [10,11].

Our patient developed critical limb ischemia of his right foot within a few days of admission, with milder ischemic symptoms in his other three limbs. The diagnosis of PAN was speculated due to the multi-system presentation, with negative ANCA and lack of response to antibiotic therapy. At the time, there was no confirmed histology to support the diagnosis. However, due to clinical urgency of the limb-threatening critical ischemia, the decision was made to proceed to immunosuppression. He was immediately commenced on high-dose steroids and cyclophosphamide within 48 hours of the rheumatology consultation.

Angiography and CT angiography showed vascular occlusion of several vessels but did not find any microaneurysms that are typically seen in PAN. We also failed to demonstrate any of the typical histopathological findings of PAN on the muscle biopsy. This was perhaps less surprising because the patient had commenced treatment with high-dose corticosteroids and cyclophosphamide at least three weeks prior to the biopsy being obtained, so the vasculitis was partially treated.

A recent systematic review highlighted that in cases of PAN with peripheral neuropathy, a combined nerve and muscle biopsy detects vasculitic lesions more frequently than muscle biopsy alone, thereby enhancing the sensitivity of histopathological diagnosis [12].

Induction treatment for PAN with organ or life-threatening disease is IV pulsed corticosteroids, typically methylprednisolone 500-1000mg/day over three to five days alongside intravenous cyclophosphamide [13]. Cyclophosphamide was chosen as induction therapy rather than rituximab, as it was considered more rapidly acting in this setting. Our patient received IV immunoglobulins too, as there were features of myositis.

Once clinical remission of PAN is achieved, the treatment regimen usually includes a tapered reduction of glucocorticoids and a steroid-sparing agent such as methotrexate, azathioprine or mycophenolate mofetil. Given the background of autoimmune hepatitis in long-term remission and normal baseline liver function, we plan to start mycophenolate mofetil (MMF) for maintenance therapy, as the patient had previously developed PAN while on azathioprine. Our rheumatology team has experience co-managing patients with hepatology teams using MMF for autoimmune hepatitis.

If there is a relapse and the presentation appears refractory, there is literature to suggest considering genetic testing for adenosine deaminase 2 (ADA2) deficiency. This condition mimics PAN and often presents with ischemic stroke and responds more favourably to tumour necrosis factor inhibitor treatment [12].

Lastly, it is interesting to note that seven years previously, the patient had required treatment with corticosteroids and cyclophosphamide for a membranous glomerulonephritis (MGN). This was felt to be primary at the time. A review of the literature reveals several reports describing the concurrent occurrence of PAN and MGN as extrahepatic manifestations of chronic HBV infection [14-17]. One study examining the renal histopathology of 143 renal biopsy specimens identified cases exhibiting features of PAN and MGN [18]. However, it did not elucidate any pathophysiological relationship between the two diseases. Notably, no literature was found exploring a possible link between MGN and PAN in the absence of HBV infection, leaving the potential mechanistic or causal links in non-HBV-related cases, such as the patient described in this report, unclear.

## Conclusions

This case illustrates the extensive spectrum of organ and clinical manifestations that can occur in PAN and be rapidly progressive. Our case was associated with critical lower limb ischemia, cutaneous vasculitis, orchitis, mononeuritis multiplex, myositis and infarcts affecting brain, myocardium, kidneys and spleen. It reminds us of the need to consider vasculitis as a possible diagnosis when patients present with multisystem symptoms and high inflammatory markers. There should be a high degree of suspicion for PAN and early rheumatological referral in cases of limb ischemia where typical risk factors or other features of peripheral arterial disease are absent.
